# In search of behavioral and brain processes involved in honey bee dance communication

**DOI:** 10.3389/fnbeh.2023.1140657

**Published:** 2023-06-29

**Authors:** Hiroyuki Ai, Walter M. Farina

**Affiliations:** ^1^Department of Earth System Science, Fukuoka University, Fukuoka, Japan; ^2^Laboratorio de Insectos Sociales, Departamento de Biodiversidad y Biología Experimental, Facultad de Ciencias Exactas y Naturales, Universidad de Buenos Aires, Buenos Aires, Argentina; ^3^Instituto de Fisiología, Biología Molecular y Neurociencias, CONICET-UBA, Buenos Aires, Argentina

**Keywords:** honey bee, waggle dance, brain, rhumb-line, neuromodulation, experience-based modulatory system, antenna-mechano-motor center, central complex

## Abstract

Honey bees represent an iconic model animal for studying the underlying mechanisms affecting advanced sensory and cognitive abilities during communication among colony mates. After von Frisch discovered the functional value of the waggle dance, this complex motor pattern led ethologists and neuroscientists to study its neural mechanism, behavioral significance, and implications for a collective organization. Recent studies have revealed some of the mechanisms involved in this symbolic form of communication by using conventional behavioral and pharmacological assays, neurobiological studies, comprehensive molecular and connectome analyses, and computational models. This review summarizes several critical behavioral and brain processes and mechanisms involved in waggle dance communication. We focus on the role of neuromodulators in the dancer and the recruited follower, the interneurons and their related processing in the first mechano-processing, and the computational navigation centers of insect brains.

## Introduction

The honey bee (*Apis mellifera*) is a eusocial insect that displays a complex communication system called the waggle dance (von Frisch, 1967). Incoming honey bees transpose flight information, direction, and distance of the discovered target to a small-scale walking pattern, a figure-of-eight–shaped waggle dance, on the vertical wax combs of the hive cavity (von Frisch, 1946, 1967). Through these stereotypic motor displays, honey bees share rhumb-line information, such as the location of a profitable food source or a suitable cavity for a new colony, with their hive mates. These maneuvers consist of a waggle-run phase and a return phase. In the waggle phase, oscillating air jet flows and changes in the electrostatic field caused by wagging movements with wingbeats that are produced by the dancer (Michelsen, 2003; Greggers et al., 2013; Paffhausen et al., 2021), while hive mates follow the dancer from behind and laterally (Judd, 1995; Rohrseitz and Tautz, 1999; Gil and De Marco, 2010). Immediately, the dancer continues with a return phase without any wagging movements. The occurrence and persistence of the dance displays depend on both external (e.g., food source profitability and the colony’s nutritional status) and internal stimulations (e.g., individual nutritional level and foraging experiences) of the dancers (von Frisch, 1967; Dyer, 2002; Grüter and Farina, 2009).

As part of the waggle dance itself, the round dance allows recruiting hive mates to profitable targets located within a short range of the colony (von Frisch, 1967; Gardner et al., 2008). In the round dance, the duration of the waggle phase is extremely short and contains some directional information from the hive to the target (Griffin et al., 2012). This locomotor displays change according to the distance between the hive and the feeding site; for example, increased scattering of the directions in the successive waggle runs for shorter distances and excited walking circles for closer floral patches (von Frisch, 1967). Potentially, various sensory modalities in the transmission of waggle or round dance information might be involved, such as oscillating air jet flow, substrate vibration, and electrostatic fields. However, how these signals are transferred to the brain has not been studied as thoroughly as the waggle dance itself. Analyses of the behavioral and neuronal response caused by these signals in potential foragers could improve our understanding of waggle dance communication. In addition to rhumb-line information, hive mates receive chemosensory cues of the food collected through social interactions, such as body contacts between individuals (Balbuena et al., 2012), mouth-to-mouth food transfers (Farina et al., 2005, 2007; Martínez and Farina, 2008), and the waggle dance itself (von Frisch, 1967; Dyer, 2002; Grüter and Farina, 2009; Table 1).

**TABLE 1:** External and internal factors affecting dancer and follower behaviors (reference numbers in parentheses).

	Dancer	Follower
*External factors*	Food profitability (von Frisch, 1967; Dyer, 2002; Geng et al., 2022)	Social interactions (Grüter and Ratnieks, 2011; Menzel et al., 2011; Balbuena et al., 2012)
	Colony nutrition status (Dyer, 2002; Geng et al., 2022)	Colony nutrition status (Dyer, 2002; Biesmeijer and Seeley, 2005; Farina et al., 2005, 2007; Martínez and Farina, 2008)
*Internal factors*	Foraging experiences (Dyer, 2002; Biesmeijer and Seeley, 2005; Grüter et al., 2008; Grüter and Ratnieks, 2011; Farina et al., 2020; Geng et al., 2022)	Foraging experiences (Biesmeijer and Seeley, 2005; Grüter et al., 2008; Grüter and Ratnieks, 2011; Menzel et al., 2011)
	Food wanting (Bestea et al., 2021; Huang et al., 2022)	Attention and perception neuromodulated at sensory pathway (Barron et al., 2007; Grüter et al., 2008; Grüter and Ratnieks, 2011; Menzel et al., 2011; Moauro et al., 2018; Huang et al., 2022)
		Learning and memory (BA modulated in the higher-order brain) (Hammer, 1993; Hammer and Menzel, 1995; Barron et al., 2002, 2007; Huang et al., 2022)

BA, biogenic amines; DA, dopamine; OA, octopamine; sNPF, short neuropeptide F.

The honey bee is an excellent experimental model for neuroethological study (Menzel, 2012). The complete sequencing of its genome (Honeybee Genome Sequencing Consortium, 2006) has allowed researchers to link many molecular markers with honey bee behavior. In addition, cellular high-resolution serial block-face electron microscopy (SBEM) has revealed the “connectome” of brain interneurons that are closely related to the specific behavior of insects (Hulse et al., 2021; Sayre et al., 2021). Within this framework, recent studies using new technologies have facilitated a greater understanding of the molecules and interneurons that may be involved in waggle dance communication. Our review summarizes the results of several related studies that describe current progress in the study of waggle dance communication in the honey bee, with a particular focus on the role of neuromodulators and interneurons in the brain.

## Neuromodulators driven by starvation activate appetitive responsiveness, learning, and foraging in the dancer

**FIGURE 1:**
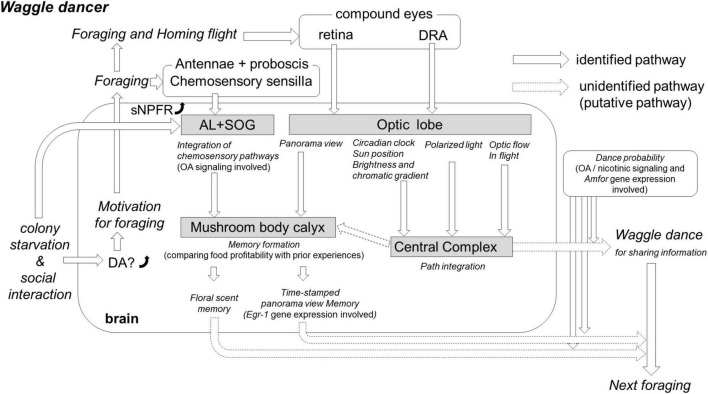
The hypothesis of the critical neurobiological processes and mechanisms involved in the waggle dance. Colony starvation enhances the dopamine (DA) level significantly in the brain of foragers (Huang et al., 2022). The high DA level affects the motivation for foraging, and putative foragers fly out to search for food. On the flower, the foragers collect food resources and simultaneously integrate this olfactory and gustatory information into both the antennal lobe (AL) and suboesphageal ganglion (SOG) through octopamine (OA) signaling (Hammer and Menzel, 1995). Colony starvation also enhances gene expression of short neuropeptide F receptors (sNPFR) in the brain (Ament et al., 2011), which enhances appetitive responsiveness and neural activities in the AL olfactory interneurons (Bestea et al., 2021). A reward-associated memory can be formed in the mushroom body (MB) calyx by comparing current food profitability with prior acquired experiences; if the current reward value surpasses the prior acquired value, the floral scent could be memorized for the next foraging bout (Menzel, 2012). In the MB calyx, a panorama view might be memorized, for instance, by performing foraging training during a time-specific procedure (e.g., time-memory; Shah et al., 2018). This time-dependent memory might be caused by expression changes in early genes (e.g., *Egr-1*) in the MB, AL, and optic lobe (OL) (Shah et al., 2018; Geng et al., 2022). Additionally, foragers acquire critical visual information around feeding sites and on the homeward flight. The retina detects information about panorama view, landmarks, and sun position that is used to memorize a profitable feeding site. The optic flow during the homeward flight, detected by the retina, allows a forager to calculate the distance to the feeder. The ommatidia of the retina dorsal rim area (DRA) detect polarized light, and their neuronal pathways reach the central complex (CX) to encode the direction from the hive. The system for encoding the direction from the hive to the feeder must be compensated by the circadian rhythm; however, its mechanism remains unknown. After returning to the hive, the incoming forager has many social interactions with nestmates that might readjust thresholds for foraging-related activities, including dance occurrence and persistency. The decision-making process to release dance is controlled by OA signaling (Barron et al., 2007); *Amfor* is the putative gene involved (George et al., 2020).

The worker honey bees monitor colony nutrition status through in-hive social interactions: the individual-to-individual trophallaxes. The trophallaxes propagate food-related information in quantitative and qualitative terms (Goyret and Farina, 2005a,b; Ramírez et al., 2010) among worker honey bees. This is possible due to the fact that food receivers might modify the feeding-related behaviors, such as gustatory responsiveness (Martínez and Farina, 2008; Ramírez et al., 2010), establish associative memory (Farina et al., 2007), and recall it (Goyret and Farina, 2005a) during trophallaxes. In a colony hunger state, the social interaction, including trophallaxes, could link individual motivational levels to forage and then to dance through critical signaling pathways in the brain (Figure 1).

In this sense, some neuromodulatory factors function in the honey bee brain as drivers of foraging-related behaviors, including the waggle dance. Within an appetitive learning context, one of the critical neuromodulatory factors is the biogenic amine octopamine (OA). Its reward value is clearly demonstrated in the octopaminergic neuron VUMmx1, which mediates the reinforcing function of reward during olfactory conditioning (Hammer and Menzel, 1995) and projects its arborization to several areas of the honey bee brain involved in cognitive processes (AL, SOG, and Mushroom body calyx, Figure 1; Hammer, 1993). OA signaling also modulates responsiveness to foraging-related stimuli as a brood pheromone (Barron et al., 2002). Barron et al. (2007) suggested that treatment with OA enhanced dance probability, whereas its antagonist mianserin blocked the enhancement of the round dance. OA is a critical neuromodulator that promotes foraging and the occurrence of dancing displays in active foragers.

A recent study reported another biogenic signal that functions in appetitive pathways in the honey bee brain: dopamine (DA) mediates food wanting in the bee brain (Huang et al., 2022), and DA levels in the honey bee brain differ between the starved and satiated states. Moreover, fluphenazine, a DA antagonist, blocks honey bee foraging frequency, suggesting that DA activates foraging. This recent study (Huang et al., 2022) showed that the DA level increases only during transient stages, such as the moments when successful foragers initiate their first dancing maneuvers. As the DA level decreases at the end of the waggle dance, it is speculated that DA signaling facilitates the dancer’s transient evocation of food-source properties. However, the DA levels of dance followers did not increase significantly with dance following, which suggests the dances do not activate the DA-based wanting system. Serotonin causes a similar response to DA, suggesting that the waggle dance might not activate the DA-based wanting system in followers during dancing displays. These results should be considered cautiously based on impossible DA values and time course (Taylor et al., 1992; Sasaki and Harada, 2020).

A neuropeptide called short neuropeptide F (sNPF) functions in appetitive pathways in the honey bee brain and has been found in the brains of various invertebrates, including those of the phyla Annelida and Arthropoda. During starvation, the level of sNPF increases, causing foraging-related responses in *Drosophila* (Root et al., 2011; Ko et al., 2015). In the honey bee brain, NPF gene expression is higher in foragers than in younger adults, and sNPF receptor gene expression increases and decreases with starvation and satiation, respectively, (Ament et al., 2011). This finding raises the question of whether a colony’s nutritional status affects sNPF receptor levels in hive mates involved in foraging. sNPF is regarded as a key modulator of starvation and other food-related responses because its topical application increases food consumption and appetitive responsiveness, restoring neural activities in olfactory circuits of the antennal lobe (AL, Figure 1) to the starved level (Bestea et al., 2021). In considering these related lines of evidence, we suggest that sNPF signaling in the honey bee may enhance the motivation to perform foraging-related activities and possibly the dancing display.

In addition, differences in sucrose responsiveness and the occurrence of dance have been analyzed, and changes have been reported in the expression level of the foraging gene *Amfor* associated with food search (Figure 1). Expression of the *Amfor* correlated negatively with dance activity but not with sucrose responsiveness (George et al., 2020). Additionally, sucrose responsiveness did not correlate with the intensity of dance activity under lower reward conditions, suggesting that the expression level of *Amfor* might regulate dance activity under low-reward conditions.

In summary, colony starvation increases their individual sensitivity to food-related information, induces appetitive memory, and motivates foraging and waggle dancing activities by using changes of both biogenic amine, sNPF levels, and foraging gene expression in the brain.

## Neuromodulators of the experience-based modulatory cognition system in the recruited follower

**FIGURE 2:**
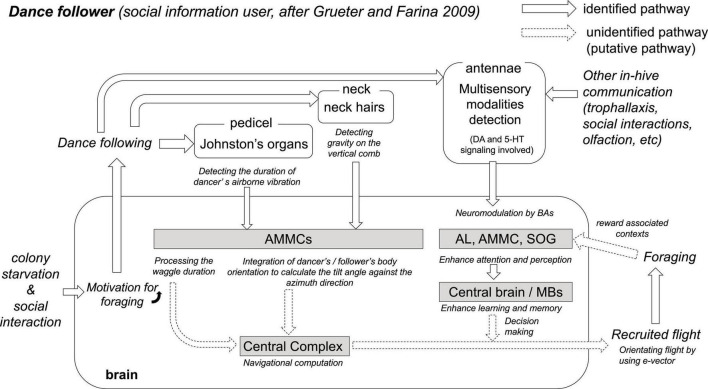
The hypothesis of critical neurobiological processes and mechanisms in the dance follower. Colony starvation and social interactions cause motivation for foraging in the brain of foragers. Putative foragers follow the dance and collect vector information via both antennae. The duration of the waggle phase is one linear parameter that indicates the distance to the food source (von Frisch, 1967). Johnston’s organs on both antennae detect the airborne vibration and electric field changes caused by dancers’ waggling and wingbeats during the waggle phase, and the sensory afferents project to the antenna mechano-motor center (AMMC, Ai et al., 2007). Interneurons in the AMMC detect the duration of the waggle dance vibration (WDV, Ai et al., 2017). At the same time, during the waggle phase, the direction from the hive to the food source is encoded in the body orientation of the dancer against the zenith on the vertical comb. Potential foragers follow the dance to decipher the direction from the hive. Neck hairs are the sensory organs for detecting gravity (the opposite direction of the zenith), and a bee can recognize its own direction relative to the zenith (Lindauer and Nedel, 1959). The sensory afferents also project into the AMMC (Ai and Hagio, 2013). Thus, the AMMC might be the processing center involved in recognizing the body orientation of the dancer against the zenith on the vertical comb. The central complex (CX) is proposed as the center for processing compass, speed, and steering information during the flight (Stone et al., 2017); however, the neural pathway from the AMMC to the CX remains unknown. The Antennae of the dance follower can also detect chemosensory and mechanosensory information. In-hive communications (e.g., dance, trophallaxis, social contact, and olfaction) upregulate the expression of biogenic amine genes (dopamine and serotonin) in the antennae (Kennedy et al., 2021). This multisensory information is transferred to the AL, AMMC, or SOG, depending on the modality of the detected stimulus. Biogenic amines (DA, OA, and 5-HT) modulate and enhance both attention and perception in AL, AMMC, and SOG, as well as learning and memory in the central brain including MBs (Mercer and Menzel, 1982; Scheiner et al., 2002; Schulz et al., 2003; Kennedy et al., 2021; Geng et al., 2022). The pathways from the above sensory and central processing to the behaviors (recruited flight and foraging) have still not been identified. However, new knowledge gained from SBEM analyses, virtual 3-dimensional brain atlas, and computational models combined with conventional neuroanatomy and neurophysiology will help to identify the missing or unidentified components in the near future (Brandt et al., 2005).

The colony hunger state and the social interactions could link individual motivational levels to follow the dances through different neuromodulating pathways in the brain (Figure 2). Thus, once a successful forager begins to display successive waggle runs, it elicits signals composed of airborne vibrations, oscillating air jet flows, substrate vibrations, electrostatic fields, and chemical release (Tautz, 1996; Michelsen, 2003; Thom et al., 2007; Grüter and Farina, 2009; Paffhausen et al., 2021). These displays not only provide spatial information to nestmates but also attract surrounding bees to the dance floor (Grüter et al., 2008). In this communication context, the prior foraging-related experiences, internal sucrose, and odor thresholds and their combination of the dance followers are highly important and promote mainly two responses: decoding the location of the feeding site transmitted by dancers or ignoring them despite performing the following trajectories behind the dancer (Table 1; Grüter and Ratnieks, 2011; Grüter et al., 2008). Additionally, the information transmitted by dancers may not be equally distributed to potential recruits (Grüter and Ratnieks, 2011): the field experienced followers tend to ignore the spatial information of the waggle dance (in 93% of all cases) and prefer to follow those dancers carrying the food odors they collected in previous foraging trips. A recent study attempted to address this issue through a network-based diffusion analysis (Hasenjager et al., 2020). The authors reported that all successful recruits to novel feeders rely on dance information, whereas dance information is relatively less important during reactivation to a known food source, and foragers are primarily guided using olfactory information. A similar analysis was used to compare the social influence of the distance information of the waggle dance during recruitment to new feeding sites (Hasenjager et al., 2022b). This approach can provide information by integrating the effect of the number of waggle runs performed with the probability of individuals subsequently arriving at a given resource. The study found little evidence that the target foraging distances (100 vs. 500 m) affected the dance followers’ responses, bringing into question how much signal performance rules affect the collecting patterns of a honey bee colony. Thus, the motivational aspects and the degree of foraging experience of the actors involved in the dancer–follower interaction are crucial to understanding the output of the receivers (Menzel, 2019). This may explain why dance followers often appear to ignore the spatial information of the waggle dance, an observation that would occur in (follower) bees with high motivation to resume foraging and that may be controlled by DA (Huang et al., 2022), which in turn could explain the prompt return to known and recently exploited feeding sites immediately after any dance display (Biesmeijer and Seeley, 2005; Grüter and Ratnieks, 2011).

The waggle dance comprises more than one informational component (redundant or non-redundant) and transfers information through more than one sensory modality (Grüter and Farina, 2009). These display patterns occur within a highly motivational context, enhance signal detection, and improve the learning of the information available to the surrounding dance followers. How dance followers receive this complex information would depend on their motivational state and attention at the internal physiological level. Thus, it is crucial to understand how often dance followers choose either to use the social information of the dance and thus decode its spatial information or to rely on previously acquired experiences about food sources they have visited in the past.

Dance followers must attend to the most relevant signal elements while filtering out less-relevant signals. To achieve this, they must possess improved sensory and cognitive abilities to acquire the encoded information of the dance. This assumption correlates well with recent studies that have focused on the behavioral and neurobiological processes underlying these communication interactions. Reportedly, the dance followings in young hive mates improve the precision of the spatial information transmitted by these individuals later in life (Dong et al., 2023). Additionally, dance followers exhibit higher responsiveness to sugared rewards and better discrimination against odorants than non-following nestmates (Moauro et al., 2018). Increased behavioral abilities should correlate with changes in neurosensory factors that drive the decision to improve the acquisition of spatial information. This assumption is consistent with a recent report that linked the use of social information in the dancing context with differences in gene expression in different parts of the honey bee nervous system (Kennedy et al., 2021). The study found that some brain areas critically involved in cognitive processes [AL; subesophageal ganglion (SOG); mushroom bodies (MB); central brain] did not vary in terms of gene expression associated with biogenic amine production between foragers that were social information users and those that used self-acquired information to resume food collection (self-acquired information users). In contrast, a considerable number of genes expressed in the antennae differed significantly between these two user types, suggesting that variability in sensory perception mediates the decision of whether or not to use social information. In addition, social information users were characterized by the upregulation of biogenic amine genes involved in the production of DA or serotonin (5-HT; Table 2; Kennedy et al., 2021). DA modulates sugar responsiveness (Scheiner et al., 2002) and affects memory retrieval in appetitive contexts (Mercer and Menzel, 1982), whereas 5-HT influences foraging activity (Schulz et al., 2003), and regulates feeding (Voigt and Fink, 2015). Honey bee foragers treated orally with OA during food collection at artificial feeders (OA-treated foragers) had fewer dance followings than control bees and increasingly used self-acquired information to collect resources. Conversely, DA-treated foragers followed more dances than control bees but did not use their social information via dances significantly, suggesting that DA might motivate bees to follow the dance but not to perceive any information (Linn et al., 2020). These results match the assumption that although OA increases the perceived sensory value of a previously experienced reward (Søvik et al., 2015), DA reduces its perception (Mercer and Menzel, 1982; Figure 2 and Table 2). An open question is the role of sNPF pathways according to their persistence (and/or motivational level) in following dances to obtain spatial information about new food sources.

**TABLE 2:** The activation of social or self-acquired information users (foragers) modulated by biogenic amines (reference numbers in parentheses).

	Social information users	Self-acquired information users
*DA*	Change levels on SRT (Scheiner et al., 2002), memory retrieval, and feeding (Mercer and Menzel, 1982)	
*5HT*	Change levels on foraging and feeding (Schulz et al., 2003)	
*OA*	Change levels on SRT (Søvik et al., 2015)	Change levels on exploration and sensory perception (Mercer and Menzel, 1982)

SRT, sucrose response threshold; DA, dopamine; 5-HT, serotonin; OA, octopamine.

It is plausible to assume that chemosensory information within the recruiting context is crucial for determining and dividing the foragers’ roles in honey bees. Food-related odor cues perceived within this context are the only signals that allow unemployed foragers to select the resource type advertised by the dancer. Recently, it was shown that food-related cues introduced into the hive by active foragers were well correlated with the foraging choices of recruits for pollen or sucrose solutions at the foraging site (Arenas et al., 2021a). These results suggest that differences in sensitivity to pollen reward divide the foragers’ roles: pollen information is mainly gained by highly pollen-sensitive hive mates that become attracted to the pollen-released cues and then become pollen recruits, and nectar information is gained by less pollen-sensitive hive mates that require strong sensory inputs, such as the taste of concentrated nectars before becoming nectar recruits (Page et al., 1995, 1998). Both recruits can switch to the other type of resource, suggesting experience-based plasticity on the foraging tendency of recruited foragers. Recently, OA signaling was found to control foraging task specialization in honey bees (Arenas et al., 2021b).

Within an appetitive learning context, OA functions as a driver of the acquisition of olfactory memory. Dance followers with prior chemosensory experience would improve on the acquisition process to decode spatial information encoded in the dance signals if the dancer is scented with a known rewarded odor. This is particularly relevant for honey bee colonies that moved to a new landscape, such as beehives used for pollination services. In-hive prior appetitive experiences might play a crucial role in the decision-making processes for both, following a dance and exploring new feeding sites. Therefore, odor-reward associations acquired inside the nest may facilitate decoding and finding the feeding sites transmitted (Balbuena et al., 2012). Similar results were reported under natural conditions, such as an agroecosystem in which hives were fed sugared syrup scented with an odorant mixture that mimicked the scent of the crop flower [for sunflower crops: (Farina et al., 2020); for apple and pear crops, (Farina et al., 2022)].

Overall, the advantage of colonies with prior odor-rewarded experiences is that there is early access to and communication of this related information within the social context of the hive. Thus, dance followers do not seem to respond only by decoding the spatial information transmitted by the dancer. Recently, it was reported that an associative memory can be established between a neutral stimulus (an odor) and antennal contact with a nestmate even though this social interaction does not imply mouth-to-mouth food exchange (Cholé et al., 2019). This result suggests that antennal contact, *per se*, could act as an appetitive reinforcement within the proper communication context through second-order conditioning. Thus, either the waggle dance, trophallactic interactions, or other mechanosensory contacts (Balbuena et al., 2012) would be sufficient to direct nest mates to the right food sources under specific contexts. Therefore, the type of information would depend on the foraging context, motivational state, prior foraging experience, and social interactions among nestmates and dancers, meaning these decision-making processes are controlled by neuromodulators in the honey bee brain.

## Interneurons and related processing in the first processing antenna-mechano-motor center of the honey bee brain

Honey bees communicate the rhumb line of a food source by encoding distance and direction within the waggle dance. During the waggle runs, a successful returning forager shakes the abdomen left and right repeatedly with intermittent wingbeats, resulting in changes in air particle movements in the near field close to the dancer (Michelsen, 2003) and in electrostatic fields in the broader area around the dancer (Paffhausen et al., 2021). These airborne and electrostatic field changes are distinguished by their characteristic frequencies and time courses, composed of two frequency components: the low-frequency domain of abdomen waggling and the high-frequency domain of wingbeats [waggle dance vibration, WDV; (Michelsen, 2003; Greggers et al., 2013; Paffhausen et al., 2021)]. This temporal pattern is composed of repetitive pulse periods, each having a constant pulse duration and inter-pulse interval (IPI, 5–25 Hz). The dance follower can detect the WDV by Johnston’s organ (JO) in the second segment of the antennae [the pedicel; (Tsujiuchi et al., 2007)]. The sensory afferents project to the dorsal lobe [i.e., the antenna-mechano-motor center, AMMC; (Ai et al., 2007)], suggesting that the AMMC is the primary vibration processing center in the bee brain (Figure 2). Comprehensive electrophysiological and neuroanatomical studies have clarified the basic neural network for WDV processing in AMMC (Ai et al., 2017). By mimicking the WDV applied to the JO, more than 100 WDV-sensitive interneurons have been identified and categorized based on their morphological characteristics.

Interneuron DL-Int-1 shows stable tonic inhibition to a train of WDV but does not maintain this tonic inhibitory response to a train of temporally modified WDV: the IPI is elongated. This finding suggests that DL-Int-1 recognizes the WDV by its temporal structure. In addition, the duration of the waggle phase linearly increases with distance to the food source, suggesting that DL-Int-1 could encode distance information in the spike signals.

Interneuron DL-Int-2 shows a tonic excitation to a train of WDV but does not maintain the tonic excitatory response to a train of temporally modified WDV; again, the IPI is elongated. The dendritic arborization of DL-Int-2 is segregated from the terminals of JO afferents in AMMC, suggesting the absence of direct synaptic connections between JO and DL-Int-2. The tonic excitation of DL-Int-2 to the train of WDV is presumed to be caused by disinhibitory excitation through DL-Int-1, which is a GABAergic inhibitory interneuron. This hypothesis is supported by computational analyses of the network model (Ai et al., 2017). DL-Int-2 has terminal arborizations in the lateral protocerebrum (LP), suggesting that the LP is at least one of the secondary centers for the WDV processing.

The direction from the hive to the food source is encoded in the body orientation of the dancer against the zenith on the vertical comb (von Frisch, 1967). For follower bees located close to the dancer, either or both antennae contact physically with the dancer. At least one temporal parameter of the antennal contact pattern would inform followers about their position relative to the dancer (Rohrseitz and Tautz, 1999; Gil and De Marco, 2010). Followers are often attracted to a dancer over distances greater than the length of a bee through either air vibration or electrostatic field changes. A follower might detect its own body orientation relative to the dancer through the timing of mechanical inputs on both sides around the dancer (Michelsen, 2003). The bilateral symmetry interneuron (bilateral DL-dSEG-LP) is one candidate for detecting the timing of mechanical inputs on both sides in followers. The bilateral DL-dSEG-LP responds to each pulse in WDV applied from both sides of JO with on-phasic excitation (Ai et al., 2017). The delay of on-phasic excitation is constant and less than 10 ms independent of pulse duration and IPI, suggesting that this neuron only detects the timing of the mechanical signal onset and does not recognize the temporal structure of the WDV.

As mentioned above, a follower detects body orientation against the zenith on the vertical comb during the waggle phase and translates it into the direction from the hive to the food source location. Neck and abdomen hairs are candidate sensory organs for detecting gravity (the opposite direction of the zenith), and a bee can recognize its own direction against the zenith (Lindauer and Nedel, 1959; von Frisch, 1967). The sensory afferents of neck hairs project into and closely terminate in the AMMC, as well as those of JO (Ai and Hagio, 2013), suggesting that unknown interneurons in the AMMC might integrate mechano-sensory inputs with gravity proprioception to recognize the body orientation of the dancer against the zenith on the vertical comb.

## Key interneurons and related processing in the central complex of the insect brain

After following a dance, recruits perform a straight flight in the direction along the acquired polar flying instruction (rhumb line) in the first phase of the foraging flight (Riley et al., 2005; Menzel et al., 2011). During the foraging flight, the recruits integrate the polar flight instruction with visual information (e.g., celestial cues such as the sun, polarization pattern, brightness, and chromatic gradient) and the optic flow to detect the speed and angular velocity during flight. This visual information received by compound eyes is transferred into the central complex (CX, Figure 2; Pfeiffer and Kinoshita, 2012; Pegel et al., 2019). The CX controls flight speed through both the visual flow detected by compound eyes and the airflow detected by JO and executes orientation and navigation toward the target (Taylor et al., 2013; Currier et al., 2020; Lu et al., 2022).

Recently, the connectomes of CX in *Drosophila* and the bumble bee were revealed by using cellular high-resolution SBEM (Hulse et al., 2021; Sayre et al., 2021). The CX comprises several substructures, including the ellipsoid body (EB), protocerebral bridge (PB), noduli (NO), and fan-shaped body [FB; (Mota et al., 2011; Zeller et al., 2015)]. These connectome studies demonstrated that the neural pathway among these substructures in the CX is highly conserved among insect brains. In the next section, we combine this new information with previous work on several insect species, focusing on several key interneurons and their related processing in the CX (Table 3).

**TABLE 3:** Key interneurons and their putative-related processing in the central complex (CX).

Internal signal	External signal	Putative processing in CX	Related identified interneurons#	Related neuropiles#	Reference numbers				
					Honey bee	Bumble bee	Sweat bee	Locust	Fruit fly (*Drosophila*)
Circadian	Sun brightness and chromatic gradient	Head direction with respect to visual landmarks	EPG/PEG (CL1)	EB (CBL), PB, Gall	(Homberg, 1985; Hensgen et al., 2021; Kaiser et al., 2022)	(Sayre et al., 2021)	(Stone et al., 2017)	(Homberg et al., 2011; Bockhorst and Homberg, 2015; Pegel et al., 2018)	(Seelig and Jayaraman, 2015; Hulse et al., 2021)
Circadian	Polarized light	Map-like representation of *E*-vector. Extracting a reliable current head direction	Δ7 (TB1)	PB	(Homberg, 1985; Kaiser et al., 2022)	(Sayre et al., 2021)	(Stone et al., 2017)	(Heinze and Homberg, 2007; Homberg et al., 2011; Pegel et al., 2018)	(Hulse et al., 2021)
Self-motion	Polarized light	Angular velocity detection	PEN (CL2)	PB, EB (CBL), NO	(Hensgen et al., 2021)	(Sayre et al., 2021)		(Heinze and Homberg, 2009)	(Green et al., 2017; Turner-Evans et al., 2017; Hulse et al., 2021)
	Optic flow in flight (speed)	Navigational computation and path integration	PFN (CPU4)	PB, FB (CBU), NO	(Hensgen et al., 2021; Kaiser et al., 2022)	(Sayre et al., 2021)	(Stone et al., 2017)		(Hulse et al., 2021)
		Steering	PFL (CPU1)	PB, FB (CBU), LAL	(Homberg, 1985; Hensgen et al., 2021)	(Sayre et al., 2021)	(Stone et al., 2017)	(Homberg, 1994; Bockhorst and Homberg, 2015)	(Hulse et al., 2021)

^#^The nomenclatures of the related identified interneurons are in accordance with those of the bumble bee (Sayre et al., 2021). The parentheses in related identified interneurons and neuropils show the corresponding name used in the other insect. All related interneurons were morphologically identified in the honey bee CX (Hensgen et al., 2021; Kaiser et al., 2022). Each EPG/PEG keeps the animal’s head direction with a visual landmark in the fruit fly (Seelig and Jayaraman, 2015). Each EPG/PEG (CL1 in honey bee) has preferred azimuth angles of the unpolarized light spot (Pegel et al., 2018). Δ7s (TB1s in honey bees) are organized in map-like representation to polarized light in PB (Heinze and Homberg, 2007). PEN (CL2 in honey bees) is responsive to the rotation velocity caused by self-motions in the fruit fly (Green et al., 2017; Turner-Evans et al., 2017) and to the polarized light in locusts (Heinze and Homberg, 2009). PFN (CPU4 in honey bee) receives both optic flow signals in the NO and the current heading signals in the PB and output to the FB (Stone et al., 2017). Stone et al. (2017) speculated this neuron holds a memory signal for the home vector in the computational model. PFL (CPU1 in honey bees) sends steering signals to contralateral LAL which is the dendrite region of descending neurons to the thoracic CPG (Homberg, 1994). CBL, lower unit of central body; CBU, upper unit of central body; CL, columnar neuron; EB, ellipsoid body; FB, fan-shaped body; LAL, lateral accessory lobe; NO, noduli; PB: protocerebral bridge; TB, tangential neuron of PB; CPU, the neurons with smooth endings both in a single column of the PB and columns of the dorsal most layer of the CBU and axonal fibers with varicose endings in the LAL.

Interneuron EPG (CL1 in the honey bee) connects the EB and PB. In *Drosophila*, it has been shown that each EPG maintains the direction of the animal’s head with visual landmarks (Seelig and Jayaraman, 2015) and has preferred azimuth angles of the unpolarized light spot (Pegel et al., 2018). The EPG has been identified morphologically and physiologically in honey bees (Homberg, 1985; Hensgen et al., 2021; Kaiser et al., 2022) and might process the head direction with respect to visual landmarks.

The Δ7 (TB1 in the honey bee) interneuron connects several columns in the PB, each of which encodes the preferred angle (E-vectors) of polarized light detected through the dorsal rim area of compound eyes and serves as an internal sky compass, coding for spatial direction (Homberg et al., 2011). The Δ7 interneuron has been identified morphologically and physiologically in honey bees (Homberg, 1985; Kaiser et al., 2022) and is one of the polarized-light-based compass neurons in the sweat bee *Megalopta genalis* (Stone et al., 2017). The Δ7 interneurons are organized in a map-like representation of celestial E-vector orientations in PB (Heinze and Homberg, 2007; Bockhorst and Homberg, 2015) for steering during the foraging flight in the bees (Homberg et al., 2011).

PEN (CL2 in the honey bee) connects the PB, EB, and NO. In *Drosophila*, PEN interneurons are responsive to the rotation velocity caused by self-motions (Green et al., 2017; Turner-Evans et al., 2017), and in locust, to E-vectors of polarized light (Heinze and Homberg, 2009). In the so-called ring-attractor network (Kakaria and de Bivort, 2017; Kim et al., 2017), the information of the rotational velocity and the E-vectors are thought to be processed in recurrent connections among EB and PB through EPG neurons (from EB to PB) and PEN (from PB to EB), facilitating the angular integration of head orientation (Green et al., 2017). The morphologically analogous PEN is conserved in the honey bee CX (Hensgen et al., 2021) and might process the angular velocity detection during the foraging flight.

The PFN (CPU4 in the honey bee) interneuron connects the PB, FB, and NO. Honey bee foragers add up optic flows detected by using compound eyes and store them as an odometer during the homeward flight (Srinivasan et al., 2000). The PFN receives the input from the contralateral TN neuron, which was identified as an optic flow–sensitive speed indicator in the sweat bee *Megalopta genalis* (Stone et al., 2017). The PFN also controls orientation to airflow in the *Drosophila* (Currier et al., 2020), which is received by JO. This suggests that the PFN may receive both an optic flow signal and an airflow signal (Stone et al., 2017). Stone et al. (2017) proposed that CPU4 functioned as a direction-locked odometer.

PFL (CPU1 in the honey bee) connects the PB, FB, and lateral accessary lobe (LAL). Each PFL receives inputs from one or a few columns in both PB and FB and sends the axon terminals in the LAL, which is a motor center of command neurons. Each PFL has a preferred vector on body orientation and output steering signals to the contralateral LAL, which is the dendrite region of descending neurons to the thoracic CPG (Homberg, 1994). The analogous neuron PFL is conserved in the honey bee CX (Homberg, 1985; Hensgen et al., 2021); therefore, PFL might be a candidate interneuron to command LAL-arborized descending neurons based on the vector information encoded in the waggle dance.

The honey bee has these interneurons in the CX (Kaiser et al., 2022), which leads us to speculate that they not only have similar neural processing to the insect CX but also additional species-specific processing based on waggle dance communication. How does the duration of the waggle phase translate into the odometer during long-distance foraging navigation? How does body orientation translate into the desired heading during foraging navigation? How is the rhumb-line information acquired during the waggle dance communication stored? One candidate for the neuropile is FB. PFN, which is the candidate for rhumb-line memory, has presynaptic arborization in the FB (Stone et al., 2017). The FB also receives input from many regions of the superior protocerebrum through tangential neurons in the honey bee (Hensgen et al., 2021), and more than eight neuropeptides have been localized in *Drosophila* (Kahsai and Winther, 2011). In addition, recruited bees not only use rhumb-line information but also its conversion to cartesian map coordinates (Wang et al., 2022). In the MB calyx, the panorama view could be memorized, for instance, by performing foraging training during a time-specific procedure (e.g., time memory). The time-dependent memory might be caused by expression changes in early genes (e.g., *Egr-1*) in the MB, AL, and optic lobe (Shah et al., 2018; Geng et al., 2022). Terrain views around the feeder and the panorama which is seen when flying toward it from the hive could help the recruited bees as view-based guidances to arrive at the feeder (Cheung et al., 2014), suggesting that higher processing centers, mainly the MBs, might be involved in cognitive processes such as attention, arousal, and complex learning and memory processes.

Recent studies have shown that transposing navigation information to dance information is not a reflexive behavior, suggesting more complex memory processes (Chatterjee et al., 2019), presumably in the CX and MB, which is incorporated with other signaling systems of the honey bee, such as tremble dance, stop signals, and shaking signals (Hasenjager et al., 2022a).

## Conclusion

In this review, we have discussed some of the critical behavioral, molecular, and neurobiological processes in waggle dancers and their followers involved in searching for food resources outside the hive and for communicating food-related information into the colony (Figures 1, 2). First, using a colony starvation technique, recent studies revealed critical neuromodulators, such as DA and sNPF, that contribute to driving the waggle dancers’ outputs (Table 1). The neuromodulatory systems involved in the regulation of feeding-related behaviors might have been co-opted in the regulation of social-related behaviors such as the waggle dance. However, the cellular target(s) of the signaling involved and how honey bees control foraging remain unknown. Second, dance followers use an experience-based modulatory system for decision-making on their foraging. The system seems to be controlled by DA and OA signaling for preferences to social or self-acquired information, respectively, for foraging (Table 2). The waggle dance is part of these social repertoires and is incorporated into the complex communication network with other signaling systems and the exchange of social information in the colony. In addition, dance followers, as well as decoding spatial information, use their own prior experience (outside and inside the colony) to make a foraging-related decision. Forager bees update information from their previously-stored flight experiences to recently acquired navigation experiences to decode the spatial information of the dance. Third, SBEM analyses in the bumble bee and *Drosophila*, combined with information on previously identified interneurons in the other insects’ CX, revealed common neural circuits for processing in the CX among insect species (Table 3). The rhumb-line information received during dance followings is presumably transferred into the CX to execute foraging. In this review, we propose the existence of a critical neuropile and associated interneurons that facilitate the processing of rhumb-line information in the CX. The pathways from the AMMC to the CX have still not been identified. However, new knowledge gained from SBEM analyses, virtual 3-dimensional brain atlas, and computational models, combined with conventional neuroanatomy and neurophysiology, should help identify the missing components in the near future (Brandt et al., 2005).

To understand honey bee foraging, including waggle dance communication, it is necessary to integrate the results of comprehensive analyses of many causes at the individual level of behavior within the colony.

## Author contributions

Both authors listed have made a substantial, direct, and intellectual contribution to the work, and approved it for publication.

## References

[B1] Ai H. Hagio H. (2013). Morphological analysis of the primary center receiving spatial information transferred by the waggle dance of honeybees. *J. Comp. Neurol.* 521 2570–2584. 10.1002/cne.23299 23297020

[B2] Ai H. Kai K. Kumaraswamy A. Ikeno H. Wachtler T. (2017). Interneurons in the honeybee primary auditory center responding to waggle dance-like vibration pulses. *J. Neurosci.* 37 10624–10635. 10.1523/JNEUROSCI.0044-17.2017 28993484 PMC6596516

[B3] Ai H. Nishino H. Itoh T. (2007). Topographic organization of sensory afferents of Johnston’s organ in the honeybee brain. *J. Comp. Neurol.* 502 1030–1046. 10.1002/cne.21341 17444491

[B4] Ament S. Velarde R. Kolodkin M. Moyse D. Robinson G. (2011). Neuropeptide Y-like signalling and nutritionally mediated gene expression and behaviour in the honey bee. *Insect. Mol. Biol.* 20 335–345. 10.1111/j.1365-2583.2011.01068.x 21349120 PMC3086931

[B5] Arenas A. Lajad R. Farina W. (2021a). Selective recruitment for pollen and nectar sources in honey bees. *J. Exp. Biol.* 224:jeb.242683. 10.1242/jeb.242683 34327528

[B6] Arenas A. Lajad R. Peng T. Grüter C. Farina W. (2021b). Correlation between octopaminergic signalling and foraging task specialisation in honeybees. *Genes Brain Behav.* 20:e12718. 10.1111/gbb.12718 33251675

[B7] Balbuena M. Molinas J. Farina W. (2012). Honey bee recruitment to scented food sources: correlations between in-hive social interactions and foraging decision making. *Behav. Ecol. Sociobiol.* 66 445–452.

[B8] Barron A. Maleszka R. Vander Meer R. Robinson G. (2007). Octopamine modulates honey bee dance behavior. *Proc. Natl. Acad. Sci. U. S. A.* 104 1703–1707. 17237217 10.1073/pnas.0610506104 PMC1779631

[B9] Barron A. Schulz D. Robinson G. (2002). Octopamine modulates responsiveness to foraging-related stimuli in honey bees (*Apis mellifera*). *J. Comp. Physiol. A Neuroethol. Sens. Neural Behav. Physiol.* 188 603–610. 10.1007/s00359-002-0335-5 12355236

[B10] Bestea L. Paoli M. Arrufat P. Ronsin B. Carcaud J. Sandoz J. (2021). The short neuropeptide F regulates appetitive but not aversive responsiveness in a social insect. *iScience* 25:103619. 10.1016/j.isci.2021.103619 PMC8719019 35005557

[B11] Biesmeijer J. Seeley T. (2005). The use of waggle dance information by honey bees throughout their foraging careers. *Behav. Ecol. Sociobiol.* 59 133–142.

[B12] Bockhorst T. Homberg U. (2015). Amplitude and dynamics of polarization-plane signaling in the central complex of the locust brain. *J. Neurophysiol.* 113 3291–3311. 25609107 10.1152/jn.00742.2014 PMC4440236

[B13] Brandt R. Rohlfing T. Rybak J. Krofczik S. Maye A. Westerhoff M. (2005). Three-dimensional average-shape atlas of the honeybee brain and its applications. *J. Comp. Neurol.* 492 1–19. 10.1002/cne.20644 16175557

[B14] Chatterjee A. George E. Basu P. Brockmann A. (2019). Honey bees flexibly use two navigational memories when updating dance distance information. *J. Exp. Biol.* 222:jeb195099. 10.1242/jeb.195099 31097604

[B15] Cheung A. Collett M. Collett T. Dewar A. Dyer F. Graham P. (2014). Still no convincing evidence for cognitive map use by honeybees. *PNAS* 111 E4396–E4397. 10.1073/pnas.1413581111 25277972 PMC4210289

[B16] Cholé H. Carcaud J. Mazeau H. Famié S. Arnold G. Sandoz J. (2019). Social contact acts as appetitive reinforcement and supports associative learning in honey bees. *Curr Biol.* 29 1407–1413.e3. 10.1016/j.cub.2019.03.025 30982650

[B17] Currier T. Matheson A. Nagel K. (2020). Encoding and control of orientation to airflow by a set of *Drosophila* fan-shaped body neurons. *eLife* 9:e61510. 10.7554/eLife.61510 33377868 PMC7793622

[B18] Dong S. Lin T. Nieh J. Tan K. (2023). Social signal learning of the waggle dance in honey bees. *Science* 379 1015–1018. 10.1126/science.ade1702 36893231

[B19] Dyer F. (2002). The biology of the dance language. *Annu. Rev. Entomol.* 47 917–949. 11729095 10.1146/annurev.ento.47.091201.145306

[B20] Farina W. Arenas A. Díaz P. Susic Martin C. Corriale M. (2022). In-hive learning of specific mimic odours as a tool to enhance honey bee foraging and pollination activities in pear and apple crops. *Sci. Rep.* 12:20510. 10.1038/s41598-022-22985-5 36443327 PMC9705528

[B21] Farina W. Arenas A. Díaz P. Susic Martin C. Estravis Barcala M. (2020). Learning of a mimic odor within honey bee hives improves pollination service efficiency in a commercial crop. *Curr Biol.* 30 4284–4290.e5. 10.1016/j.cub.2020.08.018 32946747

[B22] Farina W. Grüter C. Acosta L. Mc Cabe S. (2007). Honeybees learn floral odors while receiving nectar from foragers within the hive. *Naturwissenschaften.* 94 55–60. 10.1007/s00114-006-0157-3 17021915

[B23] Farina W. Grüter C. Díaz P. (2005). Social learning of floral odours inside the honeybee hive. *Proc. Biol. Sci.* 272 1923–1928. 16191598 10.1098/rspb.2005.3172 PMC1559887

[B24] Gardner K. E. Seeley T. D. Calderone N. W. (2008). Do honeybees have two discrete dances to advertise food sources? *Anim. Behav.* 75 1291–1300. 10.1016/j.anbehav.2007.09.032

[B25] Geng H. Lafon G. Avarguès-Weber A. Buatois A. Massou I. Giurfa M. (2022). Visual learning in a virtual reality environment upregulates immediate early gene expression in the mushroom bodies of honey bees. *Commun. Biol.* 5:130. 10.1038/s42003-022-03075-8 35165405 PMC8844430

[B26] George E. Bröger A. Thamm M. Brockmann A. Scheiner R. (2020). Inter-individual variation in honey bee dance intensity correlates with expression of the foraging gene. *Genes Brain Behav.* 19:e12592. 10.1111/gbb.12592 31145838

[B27] Gil M. De Marco R. (2010). Decoding information in the honeybee dance: revisiting the tactile hypothesis. *Anim. Behav.* 80 887–894.

[B28] Goyret J. Farina W. (2005a). Non-random nectar unloading interactions between foragers and their receivers in the honeybee hive. *Naturwissenschaften* 92 440–443. 10.1007/s00114-005-0016-7 16133104

[B29] Goyret J. Farina W. (2005b). Trophallactic chains in honeybees: a quantitative approach of the nectar circulation amongst workers. *Apidologie* 36 595–600.

[B30] Green J. Adachi A. Shah K. Hirokawa J. Magani P. Maimon G. (2017). A neural circuit architecture for angular integration in Drosophila. *Nature* 546 101–106. 28538731 10.1038/nature22343 PMC6320684

[B31] Greggers U. Koch G. Schmidt V. Dürr A. Floriou-Servou A. Piepenbrock D. (2013). Reception and learning of electric fields in bees. *Proc. Biol. Sci.* 280:20130528. 10.1098/rspb.2013.0528 23536603 PMC3619523

[B32] Griffin S. Smith M. Seeley T. (2012). Do honeybees use the directional information in round dances to find nearby food sources? *Anim. Behav.* 83 1319–1324.

[B33] Grüter C. Balbuena M. Farina W. (2008). Informational conflicts created by the waggle dance. *Proc. Biol. Sci.* 275 1321–1327. 10.1098/rspb.2008.0186 18331980 PMC2602683

[B34] Grüter C. Farina W. (2009). The honeybee waggle dance: can we follow the steps? *Trends Ecol Evol.* 24 242–247. 10.1016/j.tree.2008.12.007 19307042

[B35] Grüter C. Ratnieks F. (2011). Honey bee foragers increase the use of waggle dance information when private information becomes unrewarding. *Anim. Behav.* 81 949–954.

[B36] Hammer M. (1993). An identified neuron mediates the unconditioned stimulus in associative olfactory learning in honeybees. *Nature* 366 59–63. 24308080 10.1038/366059a0

[B37] Hammer M. Menzel R. (1995). Learning and memory in the honeybee. *J. Neurosci* 15 1617–1630. 7891123 10.1523/JNEUROSCI.15-03-01617.1995 PMC6578179

[B38] Hasenjager M. Hoppitt W. Leadbeater E. (2022b). Do honey bees modulate dance following according to foraging distance? *Anim. Behav.* 184:89e97.

[B39] Hasenjager M. Franks V. Leadbeater E. (2022a). From dyads to collectives: a review of honey bee signalling. *Behav. Ecol. Sociobiol.* 76:124.

[B40] Hasenjager M. Hoppitt W. Leadbeater E. (2020). Network-based diffusion analysis reveals context-specific dominance of dance communication in foraging honeybees. *Nat. Commun.* 11:625. 10.1038/s41467-020-14410-0 32005817 PMC6994492

[B41] Heinze S. Homberg U. (2007). Maplike representation of celestial *E*-Vector orientations in the brain of an insect. *Science* 315 995–997. 10.1126/science.1135531 17303756

[B42] Heinze S. Homberg U. (2009). Linking the input to the output: New sets of neurons complement the polarization vision network in the locust central complex. *J. Neurosci.* 29 4911–4921. 10.1523/JNEUROSCI.0332-09.2009 19369560 PMC6665345

[B43] Hensgen R. England L. Homberg U. Pfeiffer K. (2021). Neuroarchitecture of the central complex in the brain of the honeybee: neuronal cell types. *J. Comp. Neurol.* 529 159–186. 10.1002/cne.24941 32374034

[B44] Homberg U. (1985). Interneurons of the central complex in the bee brain (*Apis mellifera*. L.). *J. Insect. Physiol.* 31 251–264.

[B45] Homberg U. (1994). Flight-correlated activity changes in neurons of the lateral accessory lobes in the brain of the locust *Schistocerca gregaria*. *J. Comp. Physiol. A Neuroethol. Sens. Neural Behav. Physiol.* 175 597–610.

[B46] Homberg U. Heinze S. Pfeiffer K. Kinoshita M. El Jundi B. (2011). Central neural coding of sky polarization in insects. *Philos. Trans. R. Soc. Lond. B Biol. Sci.* 366 680–687. 21282171 10.1098/rstb.2010.0199 PMC3049008

[B47] Honeybee Genome Sequencing Consortium (2006). Insights into social insects from the genome of the honey bee *Apis mellifera*. *Nature* 44 931–949. 10.1038/nature05260 17073008 PMC2048586

[B48] Huang J. Zhang Z. Feng W. Zhao Y. Aldanondo A. de Brito Sanchez M. (2022). Food wanting is mediated by transient activation of dopaminergic signaling in the honey bee brain. *Science* 376 508–512. 10.1126/science.abn9920 35482873

[B49] Hulse B. Haberkern H. Franconville R. Turner-Evans D. Takemura S. Wolff T. (2021). A connectome of the *Drosophila* central complex reveals network motifs suitable for flexible navigation and context-dependent action selection. *eLife* 10:e66039. 10.7554/eLife.66039 34696823 PMC9477501

[B50] Judd T. (1995). The waggle dance of the honey bee: which bees following a dancer successfully acquire the information? *J. Insect. Behav.* 8 343–355.

[B51] Kahsai L. Winther A. (2011). Chemical neuroanatomy of the *Drosophila* central complex: distribution of multiple neuropeptides in relation to neurotransmitters. *J. Comp. Neurol.* 519 290–315. 10.1002/cne.22520 21165976

[B52] Kaiser A. Hensgen R. Tschirner K. Beetz E. Wüstenberg H. Pfaff M. (2022). A three-dimensional atlas of the honeybee central complex, associated neuropils and peptidergic layers of the central body. *J. Comp. Neurol.* 530 2416–2438. 10.1002/cne.25339 35593178

[B53] Kakaria K. de Bivort B. (2017). Ring attractor dynamics emerge from a spiking model of the entire protocerebral bridge. *Front. Behav. Neurosci.* 11:8. 10.3389/fnbeh.2017.00008 28261066 PMC5306390

[B54] Kennedy A. Peng T. Glaser S. Linn M. Foitzik S. Grüter C. (2021). Use of waggle dance information in honey bees is linked to gene expression in the antennae, but not in the brain. *Mol. Ecol.* 30 2676–2688. 10.1111/mec.15893 33742503

[B55] Kim S. Rouault H. Druckmann S. Jayaraman V. (2017). Ring attractor dynamics in the *Drosophila* central brain. *Science* 356 849–853. 10.1126/science.aal4835 28473639

[B56] Ko K. Root C. Lindsay S. Zaninovich O. Shepherd A. Wasserman S. (2015). Starvation promotes concerted modulation of appetitive olfactory behavior via parallel neuromodulatory circuits. *eLife* 4:e08298. 10.7554/eLife.08298 26208339 PMC4531282

[B57] Lindauer M. Nedel J. (1959). Ein Schweresinnesorgan der Honigbiene. *J. Comp. Physiol. A Neuroethol. Sens. Neural. Behav. Physiol.* 42 334–364.

[B58] Linn M. Glaser S. Peng T. Grüter C. (2020). Octopamine and dopamine mediate waggle dance following and information use in honeybees. *Proc. Biol. Sci.* 287:20201950. 10.1098/rspb.2020.1950 33049176 PMC7657864

[B59] Lu J. Behbahani A. Hamburg L. Westeinde E. Dawson P. Lyu C. (2022). Transforming representations of movement from body- to world-centric space. *Nature* 601 98–104. 10.1038/s41586-021-04191-x 34912123 PMC10759448

[B60] Martínez A. Farina W. (2008). Honeybees modify gustatory responsiveness after receiving nectar from foragers within the hive. *Behav. Ecol. Sociobiol.* 62 529–535.

[B61] Menzel R. (2012). The honeybee as a model for understanding the basis of cognition. *Nat. Rev. Neurosci.* 13 758–768. 10.1038/nrn3357 23080415

[B62] Menzel R. (2019). The Waggle Dance as an Intended Flight: A Cognitive Perspective. *Insects* 10 424. 10.3390/insects10120424 31775270 PMC6955924

[B63] Menzel R. Kirbach A. Haass W. Fischer B. Fuchs J. Koblofsky M. (2011). A common frame of reference for learned and communicated vectors in honeybee navigation. *Curr. Biol.* 21 645–650. 10.1016/j.cub.2011.02.039 21474313

[B64] Mercer A. Menzel R. (1982). The effects of biogenic amines on conditioned and unconditioned responses to olfactory stimuli in the honey bee *Apis mellifera*. *J. Comp. Physiol. A Neuroethol. Sens. Neural Behav. Physiol.* 145 363–368.

[B65] Michelsen A. (2003). Signals and flexibility in the dance communication of honey bees. *J. Comp. Physiol. A Neuroethol. Sens. Neural Behav. Physiol.* 189 165–174. 12664092 10.1007/s00359-003-0398-y

[B66] Moauro M. Balbuena M. Farina W. (2018). Assessment of appetitive behavior in honey bee dance followers. *Front. Behav. Neurosci.* 12:74. 10.3389/fnbeh.2018.00074 29755329 PMC5934941

[B67] Mota T. Yamagata N. Giurfa M. Gronenberg W. Sandoz J. (2011). Neural organization and visual processing in the anterior optic tubercle of the honeybee brain. *J. Neurosci.* 31 11443–11456. 10.1523/JNEUROSCI.0995-11.2011 21832175 PMC6623125

[B68] Paffhausen B. Petrasch J. Greggers U. Duer A. Wang Z. Menzel S. (2021). The electronic bee spy: eavesdropping on honeybee communication via electrostatic field recordings. *Front. Behav. Neurosci.* 15:647224. 10.3389/fnbeh.2021.647224 33994968 PMC8115936

[B69] Page R. Jr. Erber J. Fondrk M. (1998). The effect of genotype on response thresholds to sucrose and foraging behavior of honey bees (*Apis mellifera* L.). *J. Comp. Physiol. A Neuroethol. Sens. Neural. Behav. Physiol.* 182 489–500. 10.1007/s003590050196 9565478

[B70] Page R. Jr. Robinson G. Fondrk M. Nasr M. (1995). Effects of worker genotypic diversity on honey bee colony development and behavior (*Apis mellifera* L.). *Behav. Ecol. Sociobiol.* 36 387–396.

[B71] Pegel U. Pfeiffer K. Homberg U. (2018). Integration of celestial compass cues in the central complex of the locust brain. *J. Exp. Biol.* 221:jeb171207. 10.1242/jeb.171207 29180600

[B72] Pegel U. Pfeiffer K. Zittrell F. Scholtyssek C. Homberg U. (2019). Two compasses in the central complex of the locust brain. *J. Neurosci.* 39 3070–3080. 30755489 10.1523/JNEUROSCI.0940-18.2019 PMC6468101

[B73] Pfeiffer K. Kinoshita M. (2012). Segregation of visual inputs from different regions of the compound eye in two parallel pathways through the anterior optic tubercle of the bumblebee (*Bombus ignitus*). *J. Comp. Neurol.* 520 212–229. 10.1002/cne.22776 21953619

[B74] Ramírez G. Martinez A. Fernández V. Corti Bielsa G. Farina W. (2010). The influence of gustatory and olfactory experiences on responsiveness to reward in the honeybee. *PLoS One* 5:e13498. 10.1371/journal.pone.0013498 20975953 PMC2958144

[B75] Riley J. Greggers U. Smith A. Reynolds D. Menzel R. (2005). The flight paths of honeybees recruited by the waggle dance. *Nature* 435 205–207. 10.1038/nature03526 15889092

[B76] Rohrseitz K. Tautz J. (1999). Honey bee dance communication: waggle run direction coded in antennal contacts? *J. Comp. Physiol. A Neuroethol. Sens. Neural Behav. Physiol.* 184 463–470.

[B77] Root C. Ko K. Jafari A. Wang J. (2011). Presynaptic facilitation by neuropeptide signaling mediates odor-driven food search. *Cell* 145 133–144. 10.1016/j.cell.2011.02.008 21458672 PMC3073827

[B78] Sasaki K. Harada M. (2020). Dopamine production in the brain is associated with caste-specific morphology and behavior in an artificial intermediate honey bee caste. *PLoS One* 15:e0244140. 10.1371/journal.pone.0244140 33332426 PMC7746283

[B79] Sayre M. Templin R. Chavez J. Kempenaers J. Heinze S. (2021). A projectome of the bumblebee central complex. *eLife* 10:e68911. 10.7554/eLife.68911 34523418 PMC8504972

[B80] Scheiner R. Plückhahn S. Öney B. Blenau W. Erber J. (2002). Behavioural pharmacology of octopamine, tyramine and dopamine in honey bees. *Behav. Brain Res.* 136 545–553. 10.1016/s0166-4328(02)00205-x 12429417

[B81] Schulz D. Elekonich M. Robinson G. (2003). Biogenic amines in the antennal lobes and the initiation and maintenance of foraging behavior in honey bees. *J. Neurobiol.* 54 406–416. 10.1002/neu.10138 12500315

[B82] Seelig J. Jayaraman V. (2015). Neural dynamics for landmark orientation and angular path integration. *Nature* 521 186–191. 10.1038/nature14446 25971509 PMC4704792

[B83] Shah A. Jain R. Brockmann A. (2018). *Egr-1*: a candidate transcription factor involved in molecular processes underlying time-memory. *Front. Psychol.* 9:865. 10.3389/fpsyg.2018.00865 29928241 PMC5997935

[B84] Søvik E. Perry C. Barron A. (2015). Insect reward systems: comparing flies and bees. *Adv. Insect Physiol.* 48 189–226.

[B85] Srinivasan M. Zhang S. Altwein M. Tautz J. (2000). Honeybee navigation: nature and calibration of the “odometer”. *Science* 287 851–853. 10.1126/science.287.5454.851 10657298

[B86] Stone T. Webb B. Adden A. Weddig N. Honkanen A. Templin R. (2017). An anatomically constrained model for path integration in the bee brain. *Curr. Biol.* 27 3069–3085.e11. 10.1016/j.cub.2017.08.052 28988858 PMC6196076

[B87] Tautz J. (1996). Honeybee waggle dance: recruitment success depends on the dance floor. *J. Exp. Biol.* 199 1375–1381. 10.1242/jeb.199.6.1375 9319269

[B88] Taylor D. Robinson G. Logan B. Laverty R. Mercer A. (1992). Changes in brain amine levels associated with the morphological and behavioural development of the worker honeybee. *J. Comp. Physiol. A* 170 715–721. 10.1007/BF00198982 1432851

[B89] Taylor G. Luu T. Ball D. Srinivasan M. (2013). Vision and air flow combine to streamline flying honeybees. *Sci. Rep.* 3:2614. 10.1038/srep02614 24019053 PMC3767942

[B90] Thom C. Gilley D. Hooper J. Esch H. (2007). The scent of the waggle dance. *PLoS Biol.* 5:e228. 10.1371/journal.pbio.0050228 17713987 PMC1994260

[B91] Tsujiuchi S. Sivan-Loukianova E. Eberl D. Kitagawa Y. Kadowaki T. (2007). Dynamic range compression in the honey bee auditory system toward waggle dance sounds. *PLoS One* 2:e234. 10.1371/journal.pone.0000234 17311102 PMC1794319

[B92] Turner-Evans D. Wegener S. Rouault H. Franconville R. Wolff T. Seelig J. (2017). Angular velocity integration in a fly heading circuit. *eLife* 6:e23496. 10.7554/eLife.23496 28530551 PMC5440168

[B93] Voigt J. Fink H. (2015). Serotonin controlling feeding and satiety. *Behav. Brain Res.* 277 14–31. 25217810 10.1016/j.bbr.2014.08.065

[B94] von Frisch K. (1946). Die Tänze der Bienen. *Oesterr. Zool. Zeit.* 1 1–48.

[B95] von Frisch K. (1967). *The dance language and orientation of bees.* Cambridge, MA: Harvard University Press.

[B96] Wang Z. Chen X. Becker F. Greggers U. Aalter S. Werner M. (2022). Honey bees get map coordinates from the dance. *bioRxiv* [Preprint]. 10.1101/2022.07.27.501756

[B97] Zeller M. Held M. Bender J. Berz A. Heinloth T. Hellfritz T. (2015). Transmedulla neurons in the sky compass network of the honey bee (*Apis mellifera*) are a possible site of circadian input. *PLoS One* 10:e0143244. 10.1371/journal.pone.0143244 26630286 PMC4667876

